# Modulation of H‐reflex responses and frequency‐dependent depression by repetitive spinal electromagnetic stimulation: From rats to humans and back to chronic spinal cord injured rats

**DOI:** 10.1111/ejn.14885

**Published:** 2020-07-12

**Authors:** Hayk Petrosyan, Li Liang, Asrat Tesfa, Sue A. Sisto, Magda Fahmy, Victor L. Arvanian

**Affiliations:** ^1^ Research Services Northport Veterans Affairs Medical Center Northport New York USA; ^2^ Department of Neurobiology and Behavior Stony Brook University Stony Brook New York USA; ^3^ Department of Physical Therapy Division of Rehabilitation Sciences Stony Brook University Stony Brook New York USA; ^4^ Department of Rehabilitation Science School of Public Health and Health Professions University at Buffalo Buffalo New York USA; ^5^ Physical Medicine and Rehabilitation Services Northport Veterans Affairs Medical Center Northport New York USA

**Keywords:** electromagnetic stimulation, H‐reflex, NMDA, spinal cord injury

## Abstract

The lack of propagation of signals through survived fibers is among the major reasons for functional loss after incomplete spinal cord injury (SCI). Our recent results of animal studies demonstrate that spinal electromagnetic stimulation (SEMS) can enhance transmission in damaged spinal cord, and this type of modulation depends on the function of NMDA receptors at the neuronal networks below the injury level. Here, our pilot human study revealed that administration of repetitive SEMS induced long‐lasting modulation of H‐responses in both healthy and participants with chronic SCI. In order to understand the mechanisms underlying these effects, we have used an animal model and examined effects of SEMS on H‐responses. Effects of SEMS on H‐responses, frequency‐dependent depression (FDD) of H‐reflex, and possible underlying mechanisms have been examined in both naïve and rats with SCI. Our results demonstrate that consistent with the effects of SEMS on H‐reflex seen in humans, repetitive SEMS induced similar modulation in excitability of peripheral nerve responses in both non‐injured and rats with SCI. Importantly, our results confirmed the reduced FDD of H‐reflex in SCI animals and revealed that SEMS was able to recover FDD in rats with chronic SCI. Using intraspinal injections of the NMDA receptor blocker MK‐801, we have identified NMDA receptors as an important contributor to these SEMS‐induced effects in rats with SCI. These results identify SEMS as a novel non‐invasive technique for modulation of neuro‐muscular circuits and, importantly, modulation of spinal networks after chronic SCI.

## Graphical abstract

Spinal electromagnetic stimulation modulates H‐reflex responses in healthy and chronic SCI participants. Results of our animal experiments revealed recovery of frequency dependent depression (FDD) of H‐reflex and the crucial role of NMDA receptors in SEMS induced modulation of transmission. These results identify SEMS as a novel non‐invasive technique that can be used clinically for modulation of neuro‐muscular circuits, and importantly, modulation of spinal networks after chronic SCI.

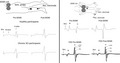

**Abbreviations**
AMPA: Alpha‐amino‐3‐hydroxy‐5‐methyl‐4‐isoxazole propionic acid
EMG: Electromyography
FDD: Frequency‐dependent depression
GABA: Gamma aminobutyric acid
IRB: Institutional Review Board
LTD: Long‐term depression
LTP: Long‐term potentiation
NMDA: N‐methyl‐D‐aspartate
SCI: Spinal cord injury
SEMS: Spinal electromagnetic stimulation
VA: Veterans administration

## 1 INTRODUCTION

Spinal cord injury (SCI) is a devastating condition that affects many important body functions of individuals. Significant deficits in transmission is one of the reasons behind functional shortfalls. Because of diminished propagation of signals through fibers that survive the injury, there is almost a complete loss of function below the injury level even after incomplete injuries. In our previous animal studies, using intracellular recordings from individual motoneurons in lumbar spinal cord, we have demonstrated that non‐invasive spinal electromagnetic stimulation (SEMS) was able to induce facilitation of synaptic transmission in damaged spinal cord by increasing function of synaptic NMDA receptors in chronic SCI animal model (Hunanyan, Petrosyan, Alessi, & Arvanian, 2012). Importantly, when SEMS administration was combined with exercise it induced sustained recovery of function in rats with chronic contusion SCI (Petrosyan, Alessi, Hunanyan, Sisto, & Arvanian, 2015).

Electromagnetic stimulation applied at spinal levels has been in broad clinical use as a non‐invasive method to examine transmission and provides a valid assessment of preserved circuits (Ugawa et al., 1989; Curt, Keck, & Dietz, 1998; Matsumoto et al., 2010; Gerasimenko et al., 2010; Petersen *et al.,* 2012; Knikou, 2013; Sasada et al., 2014; Beaulieu & Schneider, 2015). Modulation of functions with SEMS has been reported in various studies (Bycroft, Craggs, Sheriff, Knight, & Shah, 2004; Fujishiro et al., 2002; Yamanishi et al., 2000). Spinal electromagnetic stimulation has been shown to reduce spastic tone in SCI patients when applied at lumbar levels (Krause, Edrich, & Straube, 2004) and decrease spasticity in patients with multiple sclerosis (Nielsen, Sinkjaer, & Jakobsen, 1996).

Even though reported effects of electromagnetic stimulation applied at spinal levels are significant and this technique has substantial therapeutic potential, the mechanisms underlying these effects remain understudied and require investigation using animal models. Here, we present results of our pilot study investigating effects of SEMS on H‐reflex in non‐injured and people with chronic SCI. The H‐reflex represents a reliable measure of neurophysiological properties at spinal circuitry that is distorted following SCI and has been an important electrophysiological method to evaluate changes occurring at spinal level in people with SCI and animal models (Thomson et al., 1992, 1998). The major purpose of this study was to examine underlying mechanisms of SEMS‐induced effects on H‐reflex using animal model of chronic contusion SCI. Particularly, we have examined whether NMDA receptors reported to be involved in SEMS‐induced facilitation of transmission in damaged spinal cord (Hunanyan et al., 2012) are similarly involved in SEMS‐induced effects on H‐reflex.

Preliminary results of this study have been reported in abstract form (Petrosyan et al., 2019).

## 2 Material and Methods

### 2.1 Participants

Four participants with chronic spinal cord injury (SCI) and twelve healthy adult volunteers with no known history of neurological disorders participated in the study at the Northport VA Medical Center and Stony Brook University. All participants were screened for inclusion/exclusion criteria (see below), gave written informed consent to participate, and completed up to 3 sessions (separated for at least one day). Mean age of participants was 77 ± 3.8 years for SCI and 57.7 ± 7.5 years for healthy participants. SCI participants were all male veterans, and healthy participants included eight males and four females. All SCI participants had some voluntary movement of their legs and were grade D on the ASIA Impairment Scale (AIS D), see Table 1 for details. Participants were excluded from the study if they had any metal implants, any implanted electrical devices, any medications that could raise seizure threshold, or any cardiac conditions. All females were provided a pregnancy test where negative results allowed participation. This study was approved by the local Institutional Review Board (IRB) at Stony Brook University and Northport VA Medical Center and was conducted in accordance with the Declaration of Helsinki.

**Table 1:** Demographics and characteristics of participants with spinal cord injury

Gender	Age	Level of Injury	AIS	Cause of injury	Years post‐injury	Motor score	
						L	R
M	88	C4	D	MVA	66	19	22
M	77	C5	D	MVA	17	28	24
M	72	C3	D	MVA	21	23	26
M	71	C3	D	MVA	9	21	19

Abbreviations: AIS‐ The American Spinal Injury Association (ASIA) impairment scale, MVA‐motor vehicle accident, L‐left, R‐right.

At each session, H‐reflex and M‐wave responses were recorded with participants in prone position as baseline control measurements and SEMS was administered in the same position. After SEMS administration, H‐reflex responses were recorded again with participants in the same position to evaluate effects of SEMS on these responses.

### 2.2 Spinal electromagnetic stimulation

SEMS was administered using Magstim 200^2^ magnetic stimulator (Jali Medical, Inc. Waltham, MA) with figure‐of‐8 D70^2^ magnetic coil. Participants were placed in the prone position and spinal electromagnetic stimulation was administered at L4‐L5 spinal level. The optimal spinal level and stimulus intensity were established for each participant. The optimal spinal level and SEMS intensity were established based on motor evoked potentials (MEP) recorded from soleus muscle during which stimulus intensity was increased in 10% increments from 40% to 80% of maximum stimulator output (MSO). Stimulation intensity was set at 20% above threshold intensity required to evoke MEP in soleus muscles for each participant. The optimal parameters for each participant were used to deliver one 30‐min session of SEMS applied repetitively with 0.2 Hz frequency (totaling to 360 pulses per session). The coil was positioned with the handle toward the head for each participant to standardize procedures. A similar protocol had previously been established to strengthen transmission through damaged spinal cord to hindlimb muscles in chronic SCI animals (Hunanyan et al., 2012; Petrosyan et al., 2015).

### 2.3 Electrophysiological recordings

For peripheral nerve stimulation studies, Digitimer DS7A (Digitimer North America, LLC) constant current stimulator was used with bipolar gold‐plated bar electrode (Natus Neurology, Inc.) placed over the tibial nerve at the popliteal fossa. After finding the optimal position of stimulating probe (to evoke reproducible H‐response at minimal intensities), the probe was secured in place by tape for the entire session. The H‐reflex and M‐wave responses were evoked using 1ms duration square‐wave pulse delivered with 0.2Hz frequency. First, the threshold intensity (i.e., minimum intensity required to evoke reproducible response) was determined prior SEMS administration. Then, intensity of stimulation was adjusted to 20% above threshold intensity and maintained to compare amplitude of H‐reflex pre‐ and post‐SEMS. After measurements of H‐reflex amplitude post‐SEMS (measured using intensity at 20% of threshold pre‐SEMS), stimulus intensity has been adjusted to determine new threshold intensity of H‐reflex post‐SEMS. Electrophysiological recordings from muscles were performed using Delsys Trigno wireless EMG system (Delsys Inc.). Recording electrodes were placed on Soleus muscle at the beginning of each session and were secured in place for the entire session without removal. SEMS‐evoked responses as well as responses evoked by electric stimulation of tibial nerve were recorded via Digidata 1,440 digitizer (Molecular Devices) using Clampex 10.6 software and analyzed off‐line using Clampfit 10.6 software. Ten consecutive muscle responses were averaged, and peak‐to‐peak amplitude from the mean response was calculated for analysis.

### 2.4 Animal experiments

Animal studies were carried out on adult female Sprague‐Dawley (210 ‐ 260g body weight) rats in accordance with protocols approved by the local Institutional Animal Care and Use Committees at Stony Brook University and Northport VA Medical Center.

### 2.5 Spinal cord injuries

The animals were initially anesthetized with 3% isoflurane in 100% O_2_ and then transferred to 1.5% isoflurane in 100% O_2_ delivered via facemask to maintain anesthesia during surgeries. All procedures were performed under aseptic conditions and are described in detail in our previous studies (Petrosyan et al., 2014). Briefly, rats were given a subcutaneous injection of buprenorphine (0.01 mg/kg) before surgery and positioned on a heating pad to maintain body temperature. Dorsal laminectomy was performed to expose the T10 spinal cord level, and IH‐400 impactor device (2.5 mm tip diameter; Precision System and Instrumentation, Lexington, KY) was used to administer contusion injuries (150 Kdyn force). This injury model has been examined extensively and is proven as reliable injury model providing mild to moderate bilateral functional deficits (Metz et al., 2000; Scheff, Rabchevsky, Fugaccia, Main, & Lumpp, 2003). Locomotor deficits in rats have been evaluated using the Basso–Beattie–Bresnahan (BBB) Locomotion Score (Basso, Beattie, & Bresnahan, 1995), which is a sensitive scale for monitoring spinal cord function and functional recovery in rats and shows similar results in human ASIA lower limb and ambulatory functional scores (Metz et al., 2000). Consistent with our previous reports (Petrosyan et al., 2015), rats in this injury model exhibited a mean BBB score of (4.8 ± 0.3) at 2 days post‐injury that gradually increased and plateaued at (14.2 ± 0.5) 4 weeks post‐injury (chronic stage). During the chronic stage, rats exhibit significant deficits in stride length, base of support, and coordination, in addition to major deficits in stepping, weight supported steps, paw position during locomotion with no toe clearance. Antibiotic (Baytril 5 mg/kg) and sterile lactated Ringer's solution were administered subcutaneously as part of post‐operative care. Injections of analgesic (Carprofen 5 mg/kg) and Ringer's solution continued for 3 days following surgery and then as needed. Electrophysiological recordings were conducted at 6–10 weeks post‐initial SCI, that is, at chronic stage of SCI.

### 2.6 Spinal electromagnetic stimulation (SEMS)

Magstim Rapid^2^ (Jali Medical Inc) stimulator with 25mm figure‐eight coil was used to deliver spinal stimulation. The choice of this type of coil for the current study was based on the results demonstrating that the figure‐8 coil induces the most focused electric field with minimal spread as compared to other coils available (Deng, Lisanby, & Peterchev, 2013). This type of coil used in this study is known to provide very focal stimulation minimizing the stimulation of surrounding structures (Deng et al., 2013). Consistent with this view, our recent study revealed that responses recorded from L5 spinal level in rats and evoked by coil positioned at T2 has been abolished by spinal cord transections at L1 (Hunanyan et al., 2012). Here we used our previously reported stimulation protocol that is known to induce facilitation of motor evoked potentials in hindlimb muscles and synaptic plasticity in damaged spinal cord (Hunanyan et al., 2012). Briefly, the stimulation protocol was comprised of 4 trains with 100 pulses in each train separated by a 5 min break between trains. Stimulation pulses were delivered at 0.2Hz frequency with 100µs pulse duration. Stimulation intensity was determined for each rat and ranged from 30%–45% of maximum stimulator output (MSO) for Magstim Rapid^2^. Throughout the stimulation, the coil was attached to a micromanipulator with the center of the coil positioned over the T2 spinal level and in contact with the skin. This stimulation protocol was shown to induce a long‐lasting facilitation of transmission in spino‐muscular circuitry in rats with chronic SCI (Petrosyan et al., 2015; Petrosyan, Alessi, Sisto, Kaufman, & Arvanian, 2017).

### 2.7 Electrophysiological recordings of H‐reflex and M‐wave

Electrophysiological experiments were performed under ketamine (80 mg/kg)/ xylazine (10 mg/kg) anesthesia (intraperitoneal injections). Rats were placed on a heating pad to maintain body temperature during recordings; heart rate and temperature were monitored continuously throughout the experiment. One‐fifth dose of ketamine/xylazine mixture was administered intramuscularly as needed during experiment to supplement anesthesia. Rats were placed on a heating pad to maintain body temperature during recordings; heart rate and temperature were monitored continuously throughout the experiment. The peripheral stimulation and recordings of H‐reflex were performed from hindlimb muscles as previously described (D’Amico et al., 2011). Briefly, for recording of H‐reflex one insulated fine wire electrode, with ~ 3mm exposed and bent into sharp barb tip (0.005 mm stainless steel, A‐M Systems, Sequim, WA), was inserted into the dorsal interossei muscle through a 27G needle between the fourth and fifth metatarsals. The reference electrode was placed in the skin of the fifth digit. To elicit H‐reflex and M‐wave, the tibial nerve was stimulated at the ankle with two subcutaneous implanted electrodes at the ankle (0.005 mm stainless steel, A‐M Systems, Sequim, WA) with the cathode at the proximal position in the heel.

In one subgroup of animals, we compared this stimulation technique with direct stimulation of the tibial nerve (Figure 3) to examine possible impact of SEMS on skin conductance or surrounding tissue of the nerve. For that experiment, the tibial nerve was dissected free at the knee level and kept in a pool of mineral oil to prevent desiccation of the nerve. The stimulation was delivered using a custom‐made cuff electrode with tibial nerve mounted on it.

The stimulation was delivered using Master 9 stimulator (A.M.P.I, Israel) with A365 stimulus isolator (WPI) at 0.2Hz ‐ 10Hz frequency (depending on the protocol) with 0.1 ms pulse duration. Muscle responses were amplified, filtered, and recorded for off‐line analysis using pClamp 10 software (Molecular Devices).

For investigation of involvement of NMDA receptors in SEMS‐induced effects in a subgroup of rats, we performed intraspinal injections of MK‐801 (Sigma‐Aldrich), a non‐competitive blocker of NMDA receptors, into spinal cord during electrophysiological recordings as previously described (Hunanyan et al., 2012). Briefly, for a subgroup of animals, dorsal laminectomy was performed to expose L3‐L4 spinal cord level (corresponding to location of motoneurons involved in generation of recorded H‐reflex) prior to the H‐reflex baseline measurements. MK‐801 injections (0.3 µl per injection, 100 µM) were made using pulled glass micropipettes via a Hamilton syringe attached to a micromanipulator.

### 2.8 Statistical analysis

Statistical analyses were performed using SigmaPlot 11.0 software (Systat Software). Paired *t* test or *t* test was used to examine effects of SEMS and compare the groups. Mann–Whitney rank sum or Wilkinson signed rank tests were performed as needed based on data distribution. Data are presented as means ± SE, and *p* < .05 was considered statistically significant.

## 3 RESULTS

### 3.1 Human pilot study

#### 3.1.1 Effects of SEMS on H‐reflex responses

**Figure 1:**
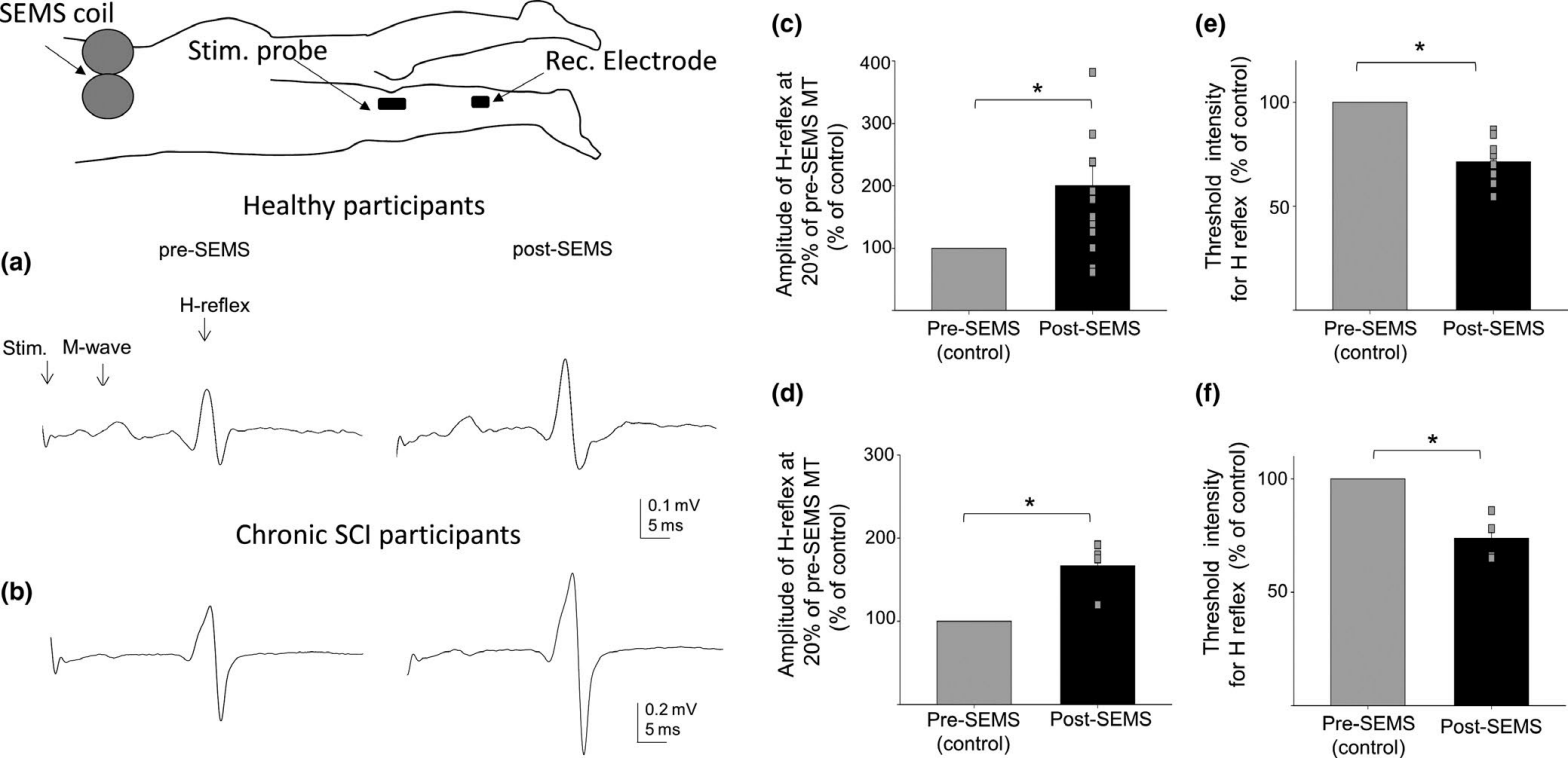
SEMS administration induced facilitation of H‐reflex responses in healthy and chronic SCI participants. (a, b) Representative averaged traces of H‐reflex and M‐wave responses recorded from the soleus muscle from one healthy (a) and one chronic SCI (b) participant, pre‐ and post‐SEMS administration and evoked with same current intensities (20% above motor threshold pre‐SESM for each individual). (c, d) Summary of results demonstrating significant increase in the amplitude of H‐reflex after SEMS administration in healthy (C) and chronic SCI (D) participants. (e, f) Summary of results demonstrating change in the threshold intensity (stimulation current intensity required to elicit the minimal H‐reflex) after SEMS administration in healthy (e) and chronic SCI (f) participants. Summary of results is presented as percent change of control for each participant, where control (pre‐SEMS) represents 100%. Diagram illustrates the position of participants, the location of SEMS coil, the location of stimulation electrode for tibial nerve, and recording electrode for H‐reflex and M‐wave from soleus muscle. (*n* = 12 healthy and *n* = 4 SCI participants; * *p* < .05 pre‐ versus post‐SEMS)

In this pilot study, we have examined the effects of repetitive SEMS on amplitude and threshold intensity of H‐reflex responses recorded in response to electric stimulation of tibial nerve in healthy and chronic SCI participants. Our results revealed that a single train of SEMS administration resulted in facilitation of H‐reflex amplitude in both healthy and SCI participants. Results summarizing the effects of SEMS on H‐reflex in humans is presented in Figure 1. Representative traces of H‐reflex and M‐wave responses recorded from one healthy (Figure 1a), and one SCI (Figure 1b) participant pre‐ and post‐SEMS administration is presented. The amplitude of H‐reflex, evoked at the same current intensity (20% above threshold pre‐SEMS) after SEMS administration, was greatly increased compared to pre‐SEMS measurements (Figure 1a, b). Stimulation intensity used for this comparison was determined for each participant and calculated as 20% above threshold stimulation intensity required to elicit H‐reflex prior SEMS administration. This stimulus intensity was maintained to compare amplitude of H‐reflex pre‐ and post‐SEMS. Summary of results for all participants demonstrate significant facilitation of H‐reflex responses post‐SEMS. The amplitude of H‐reflex was increased to 200 ± 36% compared to pre‐SEMS amplitudes evoked at same current intensities (control measurements were used as 100% for each participant individually) for healthy participants (Figure 1c, *n* = 12, *p* < .05) and increased to 166 ± 15% for SCI participants (Figure 1d, *n* = 4, *p* < .05). The facilitation of H‐reflex amplitude was also associated with a change in threshold intensity of stimulation current required to evoke H‐reflex. Threshold intensity required to elicit H‐reflex was significantly decreased in both healthy and SCI participants (Figure 1e, f). Mean threshold intensity to evoke H‐reflex was 71 ± 2.7% post‐SEMS compared to pre‐SEMS measures (control measurements were used as 100% for each participant individually) for healthy participants (Figure 1e, *n* = 12, *p* < .05) and 73 ± 5% post‐SEMS for SCI participants (Figure 1f, *n* = 4, *p* < .05). These results demonstrate the modulatory effects of SEMS on H‐reflex responses in both SCI individuals and individuals with no neurological deficits.

### 3.2 Animal studies

#### 3.2.1 Effects of SEMS on H‐reflex responses

Following the results obtained in the human pilot study presented above, we conducted broad evaluation of the effects of SEMS on H‐reflex responses in non‐injured and in rats with chronic SCI and examined possible mechanisms underlying SEMS‐induced effects. Comparison of amplitude of H‐reflex responses in non‐injured and rats with SCI prior to SEMS administration revealed that the amplitude of H‐reflex in general was significantly larger in rats with chronic SCI compared to non‐injured animals. Mean amplitude of H‐reflex (evoked at 20% above H‐reflex threshold intensity) in non‐injured rats was 0.37 ± 0.13 mV (*n* = 13) compared to 0.89 ± 0.20 mV (*n* = 8) in rats with chronic SCI (*p* < .05). These results are consistent with our human results and the literature reporting larger amplitude of H‐reflex responses in humans with SCI. We have also compared the threshold intensities required to evoke H‐reflex in non‐injured rats and rats with SCI. Results revealed similar significant differences between the groups: mean threshold intensity required to elicit H‐reflex in non‐injured rats was 1.68 ± 0.14mA versus 0.58 ± 0.17 mA intensity in rats with chronic SCI (*p* < .05).

**Figure 2:**
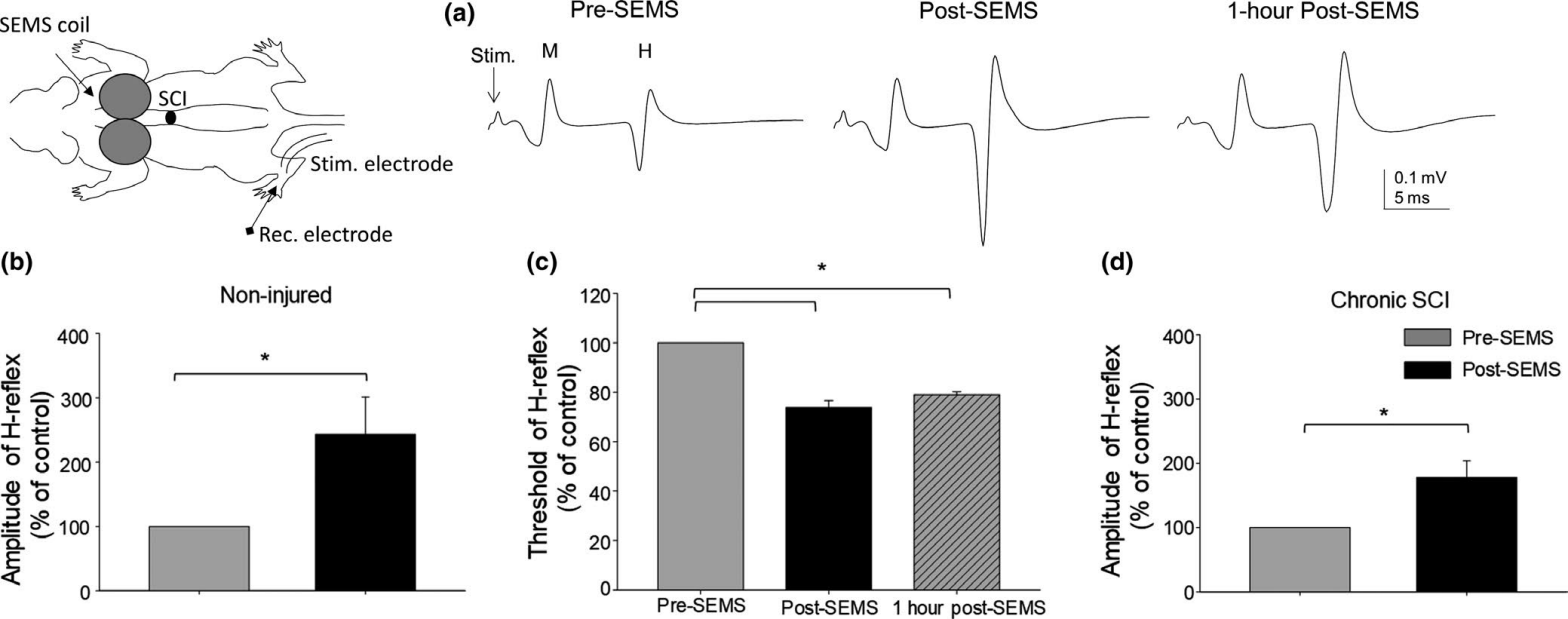
SEMS administration induced facilitation of H‐reflex responses in chronic SCI and non‐injured rats and was associated with decreased H‐reflex threshold intensities. (a) Representative traces of the H‐reflex and M‐wave responses recorded from the same non‐injured rat and evoked by subcutaneous stimulation electrodes at the ankle level at the same stimulation intensity for pre‐, post‐SEMS and 1‐hr post‐SEMS, respectively. (b) Summary of results demonstrating significant facilitation of H‐reflex amplitude pre‐ and post‐SEMS administration in non‐injured rats, respectively. (c) Summary of results demonstrating decreased threshold intensity required to evoke H‐reflex in naïve rats post‐SEMS administration and even 1‐hr post‐SEMS administration. (d) Summary of results demonstrating significant facilitation of H‐reflex amplitude pre‐ and post‐SEMS administration in rats with chronic SCI. (*n* = 13 naïve and *n* = 8 rats with chronic SCI; * *p* < .05 pre‐ versus post‐SEMS)

We further examined whether SEMS has similar effects on H‐reflex in rats as was seen in human studies. Examples of H‐reflex recordings are presented in Figure 2. Figure 2a demonstrates H‐reflex responses recorded from one naïve rat using the same stimulation current intensity pre‐ and post‐SEMS administration, respectively. The amplitude of H‐reflex responses, evoked with the same current intensity, was markedly increased after SEMS administration and remained enhanced for at least 1 hr after the termination of SEMS. In non‐injured rats, the mean amplitude of H‐reflex post‐SEMS administration increased by 143 ± 58% versus 100% control pre‐SEMS (*n* = 13; *p* < .05, Figure 2b). We also examined how SEMS administration affects the threshold intensity required to evoke H‐reflex. Our results showed that, consistent with the results of human studies (Figure 1), facilitation of H‐reflex post‐SEMS application in animals was associated with a decrease in threshold intensities required to evoke H‐reflex responses (Figure 2c). The mean threshold intensity for H‐reflex was 73.8 ± 2.7% post‐SEMS compared to pre‐SEMS control (100%) (Figure 2c, *p* < .05, *n* = 5). In the same rats, we examined the time‐course of SEMS‐induced facilitation of H‐reflex responses. We examined whether the effect of SEMS is long lasting. We measured H‐reflex immediately after SEMS and 1‐hr post‐SEMS and compared these responses to H‐reflex responses recorded before SEMS application to determine how long the observed threshold changes were sustained. The threshold intensity required to evoke H‐reflex was still significantly lower 1‐hr post‐SEMS administration; 79 ± 1.1% post‐SEMS compared to 100% pre‐SEMS (Figure 2c, *n* = 5; *p* < .05).

**Figure 3:**
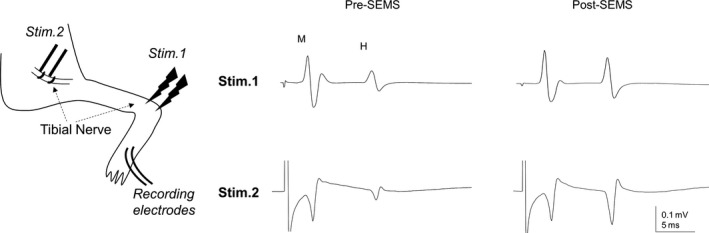
Direct stimulation of tibial nerve revealed similar facilitation of H‐reflex responses post‐SEMS. Representative traces of H‐reflex recordings evoked by subcutaneous stimulation electrodes at the ankle level (Stim.1; as in Figure 2 and following figures) and direct stimulation of tibial nerve at the knee level (Stim.2) in the same rat pre‐ and post‐SEMS, respectively. Diagram illustrates two stimulation protocols and recording electrodes used in this study

It was important to examine if possible SEMS‐induced changes in conductance of the skin or blood flow in the surrounding tissue could perhaps affect the efficacy of tibial nerve electric stimulation and activation of the nerves. Therefore, in one subgroup of animals we examined whether the electrical stimulation protocol used in this study, that is, subcutaneous implantation of electrodes (see methods), could have any possible effects on obtained results. We performed experiments where in the same rats (*n* = 4), we have recorded H‐reflex responses using same recording electrodes, but evoked by two methods of electric stimulation: (1) using regular stimulation protocol, that is, subcutaneous implantation of stimulation of electrodes at the ankle level and (2) by direct activation of tibial nerve with cuff electrode (see methods, Figure 3). Results revealed that after SEMS administration H‐reflex responses evoked through both stimulation protocols showed similar facilitation (*n* = 4, Figure 3). Facilitation of H‐reflex amplitude using our stimulation protocol (1) was 186 ± 29% compared to 208 ± 33% facilitation using stimulation protocol (2) (*n* = 4, *p* > .05). These results demonstrate that modulation of H‐reflex responses by SEMS is apparently a result of changes in the excitability at the spino‐muscular circuitry rather than other peripheral effects. In addition, these results provide additional evidence supporting the stimulation protocol used in this and many other studies (Lee, Johnson, & Wrathall, 2007; D’Amico et al., 2011) as a reliable stimulation protocol for H‐reflex recordings in animal models.

In rats with chronic SCI, SEMS administration resulted in a similar facilitation of H‐reflex responses. In rats with chronic SCI post‐SEMS administration, the amplitude of H‐reflex increased by 77 ± 26% versus 100% control pre‐SEMS (*n* = 5; *p* < .05, Figure 2d). In rats with chronic SCI, the threshold intensity for H‐reflex was decreased post‐SEMS in a similar fashion as in naïve rats, the mean threshold intensity for H‐reflex was 76.4 ± 1.7% post‐SEMS administration compared to pre‐SEMS control (100%) (*p* < .05, *n* = 5). These results demonstrate that SEMS is having similar modulatory effect in animal model as in humans described above.

#### 3.2.2 Effects of SEMS on frequency‐dependent depression of H‐reflex

Next, we examined how SEMS‐induced facilitation affects the frequency‐dependent depression (FDD) of H‐reflex. We measured the magnitude of FDD of the H‐reflex in non‐injured and chronically injured rats.

**Figure 4:**
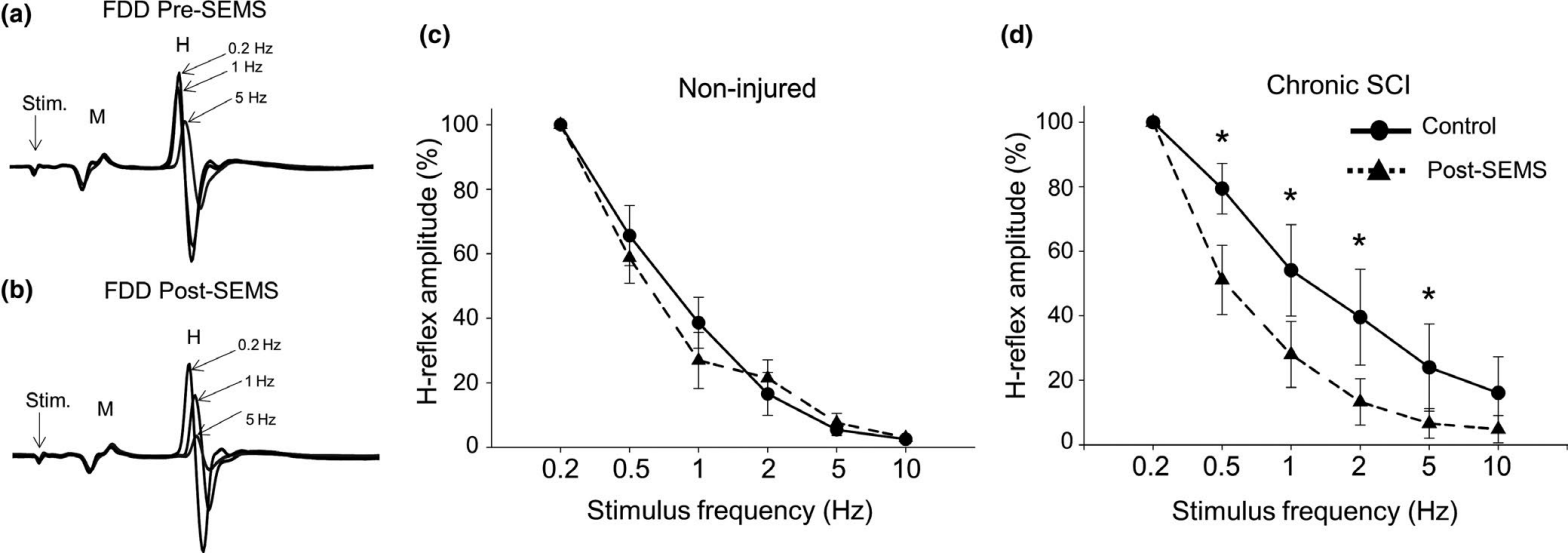
SEMS administration restores frequency‐dependent depression (FDD) of H‐reflex in rats with chronic SCI. (a, b) Superimposed traces of H‐reflex responses recorded from same SCI animal at different stimulation frequencies (0.2, 1, 5Hz) before (a) and after (B) SEMS administration, respectively. (a) Pre‐SEMS H‐reflex amplitude in rats with chronic SCI shows slight depression with increasing the stimulation frequencies. (b) Post‐SEMS administration H‐reflex recordings from the same SCI animal demonstrate significant decrease in the amplitude of H‐reflex with increased frequencies with 1 and 5Hz stimulation. (c) Summary of results demonstrating substantial FDD prior SEMS and no significant effect of SEMS on FDD in non‐injured rats (*n* = 8, * *p* > .05, pre‐ versus post‐SEMS). (d) Summary of results demonstrating less FDD depression prior SEMS in SCI rat compared to naïve animal (as in c) and significant improvements of the FDD of H‐reflex amplitude post‐SEMS administration in rats with chronic SCI at different stimulation frequencies. 100% represents amplitude of responses recorded at 0.2Hz frequency for each rat (*n* = 6, * *p* < .05, pre‐ versus post‐SEMS)

In accordance with the literature, our results demonstrate that in rats with chronic SCI there is a major change in FDD rate, that is, significantly less depression of H‐reflex compared to control animals at the same respective frequencies (Figure 4). Figure 4a shows representative traces from one chronic SCI rat demonstrating recordings of H‐reflex at 0.2Hz, 2Hz, and 5Hz, respectively. For each rat, we have compared FDD rate before administration of SEMS and after. Importantly, after SEMS administration rats with SCI exhibit significant improvements in FDD rate (Figure 4b, d). Figure 4b demonstrates recordings of H‐reflex responses after SEMS administration for the same animal as in Figure 4a. Figure 4c and d demonstrates the summary of results presenting FDD of H‐reflex at 0.2, 1, 2, 5, and 10Hz stimulations for all animals tested pre‐ and post‐SEMS administration for non‐injured control rats (Figure 4c) and rats with chronic SCI (Figure 4d). Data analysis revealed that after SEMS administration, there is a statistically significant improvement in frequency‐dependent depression of H‐reflex amplitude at 0.5, 1, 2, and 5 Hz stimulations in rats with chronic SCI. The depression of H‐reflex amplitude pre‐SEMS administration for rats with SCI was 79.4 ± 7.8% at 0.5Hz, 54 ± 14.1% at 1, 39.5 ± 14.8% at 2, and 23.9 ± 13.5% at 5Hz; post‐SEMS administration same rats demonstrated 51.1 ± 10.7% at 0.5Hz, 28 ± 10.2% at 1, 13.3 ± 7.1% at 2, and 6.6 ± 4.5% at 5Hz, respectively (Figure 4d, *p* < .05, *n* = 6). Our results also revealed that there is no significant change in the rate of FDD of H‐reflex in non‐injured animals after SEMS administration (65.6 ± 9.2% at 0.5Hz, 38.6 ± 7.8% at 1, 16.5 ± 6.6% at 2, 5.4 ± 1.7% at 5Hz and 2.4 ± 1.3% at 10Hz, pre‐SEMS versus 58.7 ± 7.8% at 0.5Hz, 26.9 ± 8.6% at 1, 21.4 ± 5.7% at 2, 7.5 ± 2.9% at 5Hz, and 2.9 ± 0.5% at 10Hz post‐SEMS administration, respectively) (Figure 4c, *p* > .05). These results demonstrate potent effects of SEMS on modulation of properties of H‐reflex responses in chronically injured spinal cord.

#### 3.2.3 The role of NMDA receptors

**Figure 5:**
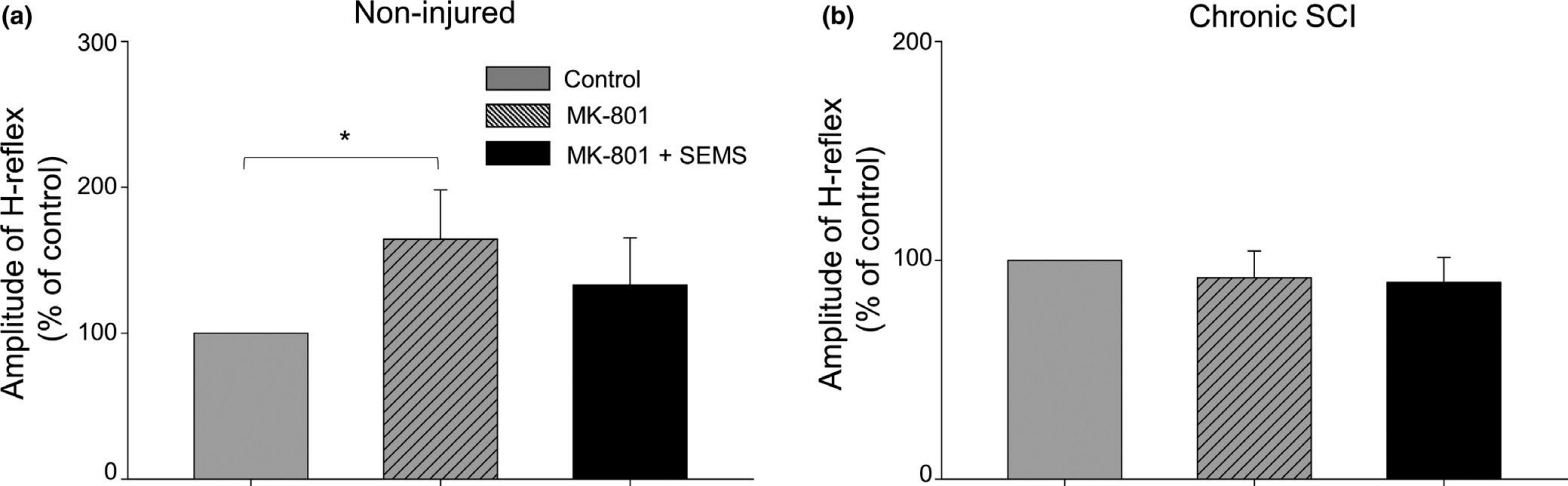
Significant role of NMDA receptors in SEMS induced modulation of H‐reflex responses. (a) Summary of results demonstrating changes in H‐reflex amplitude in non‐injured rats before, after intraspinal injections of MK‐801 and after further SEMS administration following MK‐801 injections in same rats. Intraspinal injections of MK‐801 significantly increased the amplitude of H‐reflex in non‐injured rats; further SEMS administration did not induce significant changes in H‐reflex responses. Note: In non‐injured rats, intraspinal injections of MK‐801 induced increase of H‐reflex amplitude and removed ability of SEMS to modulate H‐reflex amplitude. (b) Summary of results demonstrating changes in H‐reflex amplitude in rats with chronic SCI before, after intraspinal injections of MK‐801 and after further SEMS administration following the MK‐801 injections in same rats. Note: In chronic SCI, intraspinal injections of MK‐801 had no significant effect on H‐reflex amplitude (in contrast to its action in non‐injured rats), but still removed ability of SEMS to modulate H‐reflex amplitude. Results demonstrate that blocking of NMDA receptors in the spinal cord at spinal levels involved in generation of H‐reflex prevented SEMS‐induced modulation of H‐reflex responses in both non‐injured and rats with SCI. (*n* = 7 naïve and *n* = 5 rats with chronic SCI; * *p* < .05)

In order to examine the possible role of NMDA receptors in SEMS‐induced effects, we performed intraspinal injections of MK‐801 which is known to induce non‐competitive use‐dependent block of NMDA receptors in neurons (Davies et al., 1988) including motoneurons (Shanthanelson, Arvanian, & Mendell, 2009). Prior to electrophysiological measurements, a dorsal laminectomy was performed to expose L3‐L4 spinal levels (spinal levels corresponding to recording muscles) for further MK‐801 injections. After obtaining control measurements and before administration of SEMS, MK‐801 (0.3 µl) was injected intraspinally into the gray matter as previously described (Hunanyan et al., 2012). After MK‐801 injections, control measurements were taken again to evaluate any possible changes induced by injections and then SEMS protocol was administered as described above. Figure 5 shows the summary of results comparing the changes in H‐reflex amplitude after MK‐801 injections and SEMS administration following MK‐801 injections in non‐injured and rats with chronic SCI.

Following MK‐801 injections in non‐injured rats, a significant increase in the amplitude of H‐reflex responses was observed (Figure 5a). Mean amplitude of H‐reflex amplitude was increased to 164 ± 33.8% after MK‐801 injections compared to pre‐injection 100% controls (*n* = 7, *p* < .05). Importantly, SEMS was not able to induce significant changes H‐reflex amplitude when administered following MK‐801 injections (133 ± 32.2% post‐SEMS versus 164 ± 33.8% pre‐SEMS, *n* = 7, *p* > .05; Figure 5a).

In rats with chronic SCI, in contrast to non‐injured rats, injections of MK‐801 did not induce significant changes in amplitude of the H‐reflex (Figure 5b). However, consistent with the effects of MK‐801 in non‐injured rats, injections of MK‐801 in rats with SCI abolished ability of SEMS to enhance amplitude of H‐reflex (Figure 5b). In rats with chronic SCI, the mean amplitude of H‐reflex post‐MK‐801 injections was 91.9 ± 12.3% compared to 100% of control and 89.8 ± 11.3% post‐SEMS administration in the presence of MK‐801 (*p* > .05, *n* = 5: Figure 5b).

Results demonstrate that after MK‐801 injections, SEMS administration was not able to induce significant changes in amplitude of H‐reflex responses, in both chronic SCI and non‐injured rats, thus indicating that NMDA receptors are involved in SEMS‐induced effects on H‐reflex.

**Figure 6:**
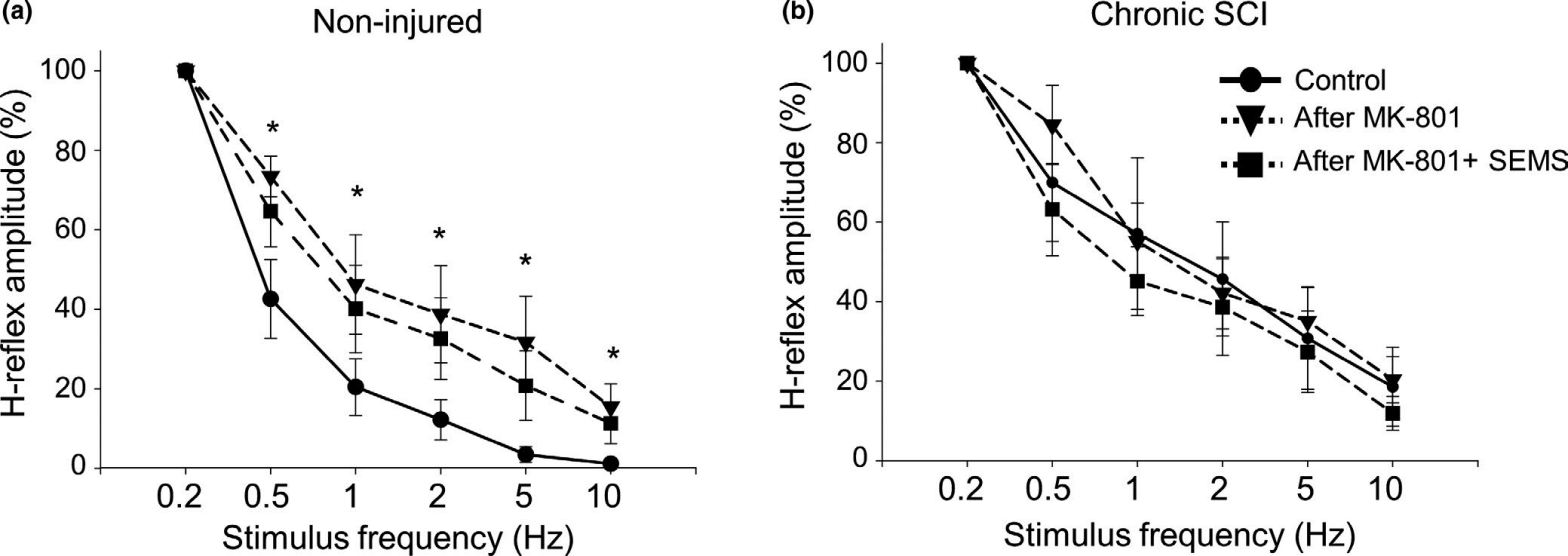
NMDA receptors play central role in the initiation of FDD of H‐reflex and the recovery of FDD induced by SEMS. (a) Summary of results demonstrating FDD of H‐reflex in non‐injured animals before, after MK‐801 injections and SEMS administration post‐MK‐801 injections in same animals, respectively. After MK‐801 injections, non‐injured rats demonstrated significant decline in the FDD of H‐reflex; further SEMS administration after MK801 injections did not induce significant changes in FDD of H‐reflex in non‐injured rats. (b) Summary of results demonstrating FDD of H‐reflex in rats with chronic SCI before, after MK‐801 injections and SEMS administration post‐MK‐801 injections in same animals, respectively. No significant changes in the FDD of H‐reflex were observed after MK‐801 injections, and similarly no significant changes were observed after SEMS administration in the presence of MK‐801. Note: Intraspinal injections of MK‐801 removed ability of SEMS to recover FDD in chronic SCI animals. (*n* = 7 naïve and *n* = 4 rats with chronic SCI; * *p* < .05)

Further, we have examined whether NMDA receptors are similarly involved in SEMS‐induced effects on FDD of H‐reflex in rats with SCI described above. We measured the magnitude of FDD of H‐reflex before and after intraspinal injections of MK‐801, and then again after administration of SEMS in the same rats. In non‐injured animals, intraspinal injections of MK‐801 induced significant reduction in FDD rate of H‐reflex (Figure 6a). The amplitude of H‐reflex was 42.6% versus 64.6% at 0.5Hz stimulation, 20.4% versus 40.1% at 1Hz stimulation, 12.2% versus 32.6% at 2Hz stimulation, 3.4% versus 20.8% at 5Hz, and 1.1% versus 11.3% at 10Hz stimulation before and after intraspinal injections of MK‐801, respectively (*n* = 7, *p* < .05: Figure 6a). Further administration of SEMS following intraspinal injections of MK‐801 did not induce significant changes in the magnitude of FDD of H‐reflex (*n* = 7, *p* > .05: Figure 6a). The results obtained from non‐injured rats establish the significant role of NMDA receptors in the initiation of FDD.

In rats with SCI, intraspinal injections of MK‐801, unlike effects of MK‐801 in non‐injured rats, did not induce significant changes of FDD rate (Figure 6b). The change in H‐reflex amplitude was 69.9 versus 84.4 at 0.5Hz stimulation, 45.6% versus 42.1% at 2Hz stimulation, and 18.5% versus 20.3% at 10Hz stimulation, respectively (*n* = 4, *p* > .05: Figure 6b). An important finding was that intraspinal injections of MK‐801 abolished the ability of SEMS to recover FDD in rats with SCI. Results demonstrate that administration of SEMS following intraspinal injections of MK‐801 did not induce significant changes in the magnitude of FDD of H‐reflex. The amplitude of H‐reflex was 84.4% versus 63.1% at 0.5Hz stimulation, 42.1% versus 38.6% at 2Hz stimulation, and 20.3% versus 11.8% at 10Hz stimulation pre‐ and post‐SEMS administration, respectively (*n* = 4, *p* > .05: Figure 6b). These results suggest that NMDA receptors are ultimately involved in SEMS‐induced recovery of FDD of H‐reflex in SCI animals. These results together demonstrate the central function of NMDA receptors in SEMS‐induced modulation in rats.

## 4 DISCUSSION

In this study, we have examined the effects of repetitive SEMS on H‐reflex responses. Our results demonstrate the ability of SEMS to modulate H‐reflex responses in healthy and SCI participants, as well as in animal models. Further, for the first time, we have provided detailed evaluation of the effects of repetitive SEMS on H‐reflex responses recorded in rats with chronic SCI. Here, we demonstrate that SEMS was able to induce long‐lasting modulation of H‐reflex responses, and most importantly, it was able to induce significant recovery of frequency‐dependent depression (FDD) of H‐reflex in rats with chronic SCI. The current study provides several new insights regarding the possible processes and mechanisms of SEMS‐induced modulation of spino‐muscular circuitry.

Results of this study provide additional evidence regarding the efficacy of SEMS. Our results from human studies revealed that SEMS induced modulation of transmission in spino‐muscular circuitry evident by recordings of H‐reflex responses. H‐reflex responses are known to be a traditional means for examining the efficacy of synaptic transmission and plasticity of the spino‐muscular system (Alrowayeh, Sabbahi, & Etnyre, 2011; Knikou, 2008; Knikou & Murray, 2019; Mezzarane & Kohn, 2002; Palmieri, Ingersoll, & Hoffman, 2004). Many studies have concluded that H‐reflex is a sensitive measure after spinal cord injuries and is affected by injury severity (Lee, Emch, Johnson, & Wrathall, 2005; Schindler‐Ivens & Shields, 2000). Any changes of H‐reflex parameters are recognized as an important neurophysiological measurement during functional recovery post‐spinal cord injuries (Ho & Waite, 2002; Thomson et al., 1998; Kocsis & Waxman, 1982).

Current results demonstrate that the rat model is a justifiable model for examination of effects of SEMS and in particular H‐reflex responses (Figure 1). The spinal cord contusion injury model in rats has been extensively examined in the literature and shown to have striking similarities to human injuries with regard to functional, electrophysiological, and morphological parameters in the chronic stage (James et al., 2011; Metz et al., 2000; Scheff et al., 2003). The validity of rat models to compare and examine possible mechanisms in relation to H‐reflex has been reported recently (Bolzoni et al., 2017). The authors reported long‐lasting modulation of H‐reflex responses following administration of transcutaneous direct current stimulation in humans and validated the results in rat model.

Results presented here demonstrating modulation of H‐reflex in SCI rats (Figure 2) are consistent with the results of our previous studies that examined transmission in spinal and spino‐muscular circuits in animals using electromyography (EMG) recordings from hind limb muscles, conducted in parallel with intracellular electrophysiological recordings from individual motoneurons and axons in lumbar spinal segments (Arvanian et al., 2009; Hunanyan et al., 2011; Petrosyan et al., 2015, 2017). These previous studies revealed diminished transmission to individual motoneurons and then to the hindlimb muscles following chronic SCI in rats. Further, the ability of SEMS to improve transmission in damaged spinal cord in animals has been reported previously (Hunanyan et al., 2012; Petrosyan et al., 2015). We have shown previously that SEMS induced facilitation of synaptic transmission in rats with chronic SCI as was evident through analysis of intracellular and extracellular recordings from lumbar spinal cord (Hunanyan et al., 2012). Facilitation sustained for at least 1 hr after stop of SEMS. Chronic administration of SEMS in animals, when combined with exercise training, resulted in recovery of function in rats with chronic SCI (Petrosyan et al., 2015).

Together, these results are consistent with other studies reporting beneficial effects of SEMS administration post‐SCI in rodent models when combined with exercise, although these studies did not include H‐reflex recordings (Ahmed & Wieraszko, 2008; Hou et al., 2014; Leydeker et al., 2013). Results of the current study demonstrate that non‐invasive SEMS induces substantial modulation of H‐reflex responses in healthy and SCI participants as well as in animal models. These results are consistent with similar results using different stimulation approaches, such as epidurally applied direct current (DC) or transcutaneous direct current stimulation (tDCS), that have recently been reported (Bolzoni et al., 2017; Jankowska et el., 2017; Hofstoetter, Freundl, Binder, & Minassian, 2018). In these studies, authors suggested that modulatory effects are due to increased axonal excitability of myelinated spinal axons as well as peripheral motor and sensory fibers. Computational modeling studies suggest that epidural stimulation, as well as transcutaneous stimulation, activates cutaneous afferents of posterior roots (Ladenbauer, Minassian, Hofstoetter, Dimitrijevic, & Rattay, 2010; Rattay, Minassian, & Dimitrijevic, 2000). Studies examining spinal electromagnetic stimulation suggest that in addition to stimulating roots, SEMS may penetrate and directly stimulate spinal cord tissue through intervertebral disc space (Efthimiadis, Samaras, & Polyzoidis, 2010). Several studies examining epidural stimulation demonstrate that through recruitment of afferent inputs electric activation can in turn involve several spinal circuits and induce activation of spinal reflex circuits, motoneuronal excitability, and pattern generating networks (Minassian *et al.,* 2007; Sayenko, Angeli, Harkema, Edgerton, & Gerasimenko, 2014; Hofstoetter et al., 2015). While there are some differences between these stimulation techniques and SEMS, we do not exclude the possibility that they share similar mechanisms in the modulatory effects reported here. It is also important to note that a recent study demonstrated that proprioceptive information is critical for facilitation of functional recovery, and that epidural electrical stimulation blocks a significant amount of proprioceptive inputs in humans (Formento et al., 2018). In contrast, SEMS is suggested to activate massive proprioceptive afferents with minimal cutaneous recruitment of sensory fibers (Kunesch et al., 1993; Struppler et al., 2007). Modulating the strength of proprioceptive inputs could lead to changes in H‐reflex characteristics (Thompson & Wolpaw, 2015) and other functional effects. The underlying mechanisms of SEMS‐evoked modulation of H‐reflex in humans are yet to be determined.

As modulation of the amplitude or the threshold intensity of H‐reflex post‐SEMS administration, demonstrated in the current study, could be in response to peripheral changes, our study does not exclude this possibility. Future studies are needed to clarify peripheral versus spinal effects of SEMS administration. Current results describing modulation of FDD of H‐reflex post‐SEMS in rats with SCI, however, suggest that SEMS indeed may have spinal effects.

The functional effects of SEMS in humans have been reported. Spinal administration of electromagnetic stimulation has been reported to have beneficial effects on bladder function, urge incontinency, and bowel dysfunction post‐spinal cord injuries (Fujishiro et al., 2002; Boycroft et al., 2004; Tsai et al., 2009). Other studies demonstrated the ability of SEMS to reduce pain when combined with physical therapy (Masse‐Alarie et al., 2013, 2017). Recent study demonstrated re‐enabled voluntary bladder function in SCI individuals after SEMS administration (Niu, Bennett, Keller, Leiter, & Lu, 2018). Our current results provide additional evidence in demonstrating the similar modulatory effects of SEMS on amplitude and threshold intensity of H‐reflex in both non‐injured and rats with chronic SCI and human participants (Figures 1and 2). The results of our human study are based on a pilot study with a limited number of participants and as such presents the need to complete a study with a larger sample. We are currently conducting such a study that includes a larger number of participants with various injury severities to determine the effects of SEMS in diverse populations.

Another major finding of this current study is the effect of SEMS on frequency‐dependent depression (FDD) of H‐reflex in rats. FDD of the H‐reflex is also an important measure that provides valid assessment in damaged spinal cord. Several studies have reported FDD as an assessment of hyper‐reflexia, severity of injury and spinal disinhibition in neuropathic pain (Thomson et al., 1998; Reese et al., 2006; Lee‐Kubli & Calcutt, 2014). Our results demonstrate a significant difference in FDD rate between non‐injured and rats with chronic SCI. Non‐injured rats display consistent decrease in the amplitude of H‐reflex with higher frequencies of stimulation (Figure 4). Our results demonstrate that in rats with chronic SCI, the decrease in the amplitude of H‐reflex with increased frequencies of stimulation is less pronounced (Figure 4). These results from our animal studies are in agreement with reported results from humans demonstrating that in healthy individuals H‐reflex amplitude exhibits depression with increased frequency of stimulation, and in injured individuals FDD of H‐reflex is attenuated and less evident with increase of frequency (Calancie et al., 1993; Reese et al., 2006; Schindler‐Ivens & Shields, 2000). The ability of SEMS to modulate FDD of H‐reflex and partially reverse the lack of FDD in SCI animals (Figure 4), as demonstrated in this study, suggests a significant therapeutic potential that should be examined further in clinical studies. Such study examining effects of SEMS on FDD of H‐reflex in healthy and SCI participants is currently on going.

Our current results provide new important insights into how SEMS can induce its modulation. We identified the significant role that NMDA receptors have in SEMS‐induced effects. Here, we demonstrate that the block of NMDA receptors by intraspinal injections of MK‐801 prevents the effects of SEMS on facilitation of H‐reflex responses (Figure 5). It is important to understand the ability of MK‐801 to block function of NMDA receptors in the vicinity of its intraspinal injection site in animals under ketamine/xylazine anesthesia. Although ketamine, used in this study, is a known non‐competitive blocker of NMDA receptors, ketamine is recognized to exert a much weaker effect on function of NMDA receptors compared to MK‐801 (MacDonald et al., 1991; Vaisanen et al., 1999). Moreover, ketamine was shown to produce a greater and longer blocking effect on NMDAR channels in immature neurons, but in mature neurons ketamine showed less or minimal effect (Jin et al., 2013). The results presented here, demonstrating that the SEMS‐induced facilitation of H‐reflex responses was abolished by intraspinal injections of MK‐801, are in strong agreement with previous results demonstrating that NMDA receptors are similarly involved in SEMS‐induced modulation of synaptic transmission to individual motoneurons in lumbar spinal cord (Hunanyan et al., 2012). Further, we have demonstrated increased immunoreactivity of GluR1 and GluR2/3 receptors in rat spinal cord neurons following SEMS administration (Petrosyan et al., 2015). Trafficking of GluR receptors to the synaptic sites or away from synapses is considered to be one of the major mechanisms in modulation of synaptic strength (Hayashi et al., 2000; Luscher et al., 1999). Similarly, it has been reported before that low frequency electric stimulation can produce potentiation of spinal cord dorsal horn neurons responses and that potentiation of nociceptive neurons is dependent on NMDA receptors (Davies & Lodge, 1987; Dickenson & Sullivan, 1987). NMDA receptors are known to regulate different forms of long‐term potentiation (LTP) and long‐term depression (LTD) in CNS (Bi & Poo, 1998; D’amour & Froemke, 2015; Debanne, Gähwiler, & Thompson, 1994), including spinal cord (Arvanian et al., 2004). Function of NMDA receptors in the spinal cord synaptic network was identified as an essential component of combination treatments designed to strengthen transmission in damaged spinal cord and facilitate recovery of function after SCI (Garcia‐Alias et al., 2011). Our previous experiments revealed a critical role of NMDA receptors in the induction and maintenance of SEMS‐induced LTP‐like facilitation of synaptic responses at motoneurons pool in rats (Hunanyan et al., 2012). We found that intraspinal injections of MK‐801 had no effect on synaptic responses pre‐SEMS; however, MK‐801 injected prior SEMS removed SEMS‐induced LTP‐like facilitation of synaptic responses. Moreover, further injections of MK‐801 after SEMS administration selectively depressed the NMDA receptor‐mediated synaptic component that was facilitated as a result of SEMS administration (Hunanyan et al., 2012). These results suggest that the appearance of a MK‐801 sensitive NMDA receptor‐mediated component of motoneuron synaptic responses induced by SEMS as previously reported (Hunanyan et al., 2012), as well as the ability of MK‐801 to prevent effects of SEMS on H‐reflex responses in the current study (Figure 5), may be a result of repetitive depolarization of the motoneuron membrane at the synaptic inputs. Ability of SEMS to directly activate synaptic inputs to lumbar motoneurons in rats has been reported (Hunanyan et al., 2012). Our current results demonstrating similarity of effects of SEMS on H‐responses in rats and humans suggest that these effects may share similar mechanisms. In humans, however, considering the longer distance from the coil surface to the spinal cord and thickness of the vertebral arches, the effects of SEMS are most probably realized through activation of posterior structures entering the cord that can lead to activation of various spinal reflex circuits and circuits involved in proprioception and motoneuron excitability (Hofstoetter et al., 2015, 2018). We, however, do not exclude the possibility that effects of SEMS on the amplitude of H‐reflex in humans are mediated through modulation of excitability of fibers in stimulating nerves.

We have also identified the critical role of NMDA receptors in initiation of FDD in non‐injured animals and in SEMS‐induced recovery of FDD in rats with chronic SCI (Figure 6). Our results revealed that intraspinal injections of MK‐801 induced significant decrease of FDD rate in normal spinal cord demonstrating that NMDA receptor‐mediated transmission is essential for FDD. In fact, the FDD rate in non‐injured rats following MK‐801 intraspinal injections decreased to almost the levels evident in chronically injured spinal cord (Figure 6). These results allow us to suggest that the significant deficits in NMDA receptor transmission reported in literature following SCI could be, in part, responsible for reduction of FDD in SCI animals. We have also demonstrated for the first time that blockade of NMDA receptors attenuated the recovery of FDD induced by SEMS in SCI animals (Figure 6). Traditionally, the role of inhibitory inputs in FDD of H‐reflex has been reported. Our results, however, suggest a critical role of NMDA receptors in initiation of FDD in non‐injured animals, and in recovery of FDD by SEMS application in rats with SCI. NMDA receptors can reportedly regulate calcium ion influx in both post‐synaptic neurons and presynaptic terminals, depending on the stimulation protocol, thus resulting in depression or facilitation (Cooke & Bliss, 2006; Nevian & Sakmann, 2004; Yang & Reis, 1999). We hypothesize that the role of NMDA receptors in FDD could possibly involve the known mechanism in which activation of presynaptic NMDA autoreceptors may in turn retrogradely facilitate GABA release from the presynaptic terminals (Xue, Han, & Chen, 2010). Further studies are needed to examine and better understand correlation between inhibitory and excitatory inputs as well as pre‐ and post‐synaptic systems in the effects of SEMS. These studies are ongoing in the laboratory. In conclusion, these results provide additional pre‐clinical and clinical evidence in support of modulatory functions of SEMS.

## 5 COMPETING INTERESTS

The authors declare no conflict of interest.

## AUTHOR CONTRIBUTIONS

HP, AT, SAS, MF and VLA conceived and conducted the human experiments and data analyses; HP, LL and VLA conceived and conducted animal experiments and data analyses; HP and VLA authored the manuscript, which was edited and approved by all authors.

## Data Availability

Data are available from the corresponding author on request.

## References

[ejn14885-bib-0001] Ahmed, Z. , & Wieraszko, A. (2008). Combined effects of acrobatic exercise and magnetic stimulation on the functional recovery after spinal cord lesions. Journal of Neurotrauma, 25, 1257–1269. 10.1089/neu.2008.0626 18986227 PMC2948469

[ejn14885-bib-0002] Alrowayeh, H. N. , Sabbahi, M. A. , & Etnyre, B. (2011). Similarities and differences of the soleus and gastrocnemius H‐reflexes during varied body postures, foot positions, and muscle function: Multifactor designs for repeated measures. BMC Neurology, 11, 65 10.1186/1471-2377-11-65 21635748 PMC3146399

[ejn14885-bib-0003] Arvanian, V. L. , Schnell, L. , Lou, L. , Golshani, R. , Hunanyan, A. , Ghosh, A. , … Mendell, L. M. (2009). Chronic spinal hemisection in rats induces a progressive decline in transmission in uninjured fibers to motoneurons. Experimental Neurology, 216, 471–480. 19320005 10.1016/j.expneurol.2009.01.004 PMC2889190

[ejn14885-bib-0004] Basso, D. M. , Beattie, M. S. , & Bresnahan, J. C. (1995). A sensitive and reliable locomotor rating scale for open field testing in rats. Journal of Neurotrauma, 12, 1–21. 7783230 10.1089/neu.1995.12.1

[ejn14885-bib-0005] Beaulieu, L. D. , & Schneider, C. (2015). Repetitive peripheral magnetic stimulation to reduce pain or improve sensorimotor impairments: A literature review on parameters of application and afferents recruitment. Neurophysiologie Clinique, 45, 223–237. 10.1016/j.neucli.2015.08.002 26363684

[ejn14885-bib-0006] Bi, G. Q. , & Poo, M. M. (1998). Synaptic modifications in cultured hippocampal neurons: Dependence on spike timing, synaptic strength, and postsynaptic cell type. Journal of Neuroscience, 18, 10464–10472. 10.1523/JNEUROSCI.18-24-10464.1998 9852584 PMC6793365

[ejn14885-bib-0007] Bolzoni, F. , Esposti, R. , Bruttini, C. , Zenoni, G. , Jankowska, E. , & Cavallari, P. (2017). Direct current stimulation modulates the excitability of the sensory and motor fibres in the human posterior tibial nerve, with a long‐lasting effect on the H‐reflex. European Journal of Neuroscience, 46, 2499–2506. 10.1111/ejn.13696 28892581

[ejn14885-bib-0008] Bycroft, J. A. , Craggs, M. D. , Sheriff, M. , Knight, S. , & Shah, P. J. (2004). Does magnetic stimulation of sacral nerve roots cause contraction or suppression of the bladder? Neurourology and Urodynamics, 23, 241–245. 15098220 10.1002/nau.20009

[ejn14885-bib-0009] Calancie, B. , Broton, J. G. , Klose, K. J. , Traad, M. , Difini, J. , & Ayyar, D. R. (1993). Evidence that alterations in presynaptic inhibition contribute to segmental hypo‐ and hyperexcitability after spinal cord injury in man. Electroencephalography and Clinical Neurophysiology, 89, 177–186. 7686850 10.1016/0168-5597(93)90131-8

[ejn14885-bib-0010] Cooke, S. F. , & Bliss, T. V. (2006). Plasticity in the human central nervous system. Brain, 129, 1659–1673. 10.1093/brain/awl082 16672292

[ejn14885-bib-0011] Curt, A. , Keck, M. E. , & Dietz, V. (1998). Functional outcome following spinal cord injury: Significance of motor‐evoked potentials and ASIA scores. Archives of Physical Medicine and Rehabilitation, 79, 81–86. 9440423 10.1016/s0003-9993(98)90213-1

[ejn14885-bib-0012] D’amour, J. A. , & Froemke, R. C. (2015). Inhibitory and excitatory spike‐timing‐dependent plasticity in the auditory cortex. Neuron, 86, 514–528. 10.1016/j.neuron.2015.03.014 25843405 PMC4409545

[ejn14885-bib-0013] D'Amico, S. C. , Schuster, I. P. , & Collins, W. F. 3rd (2011). Quantification of external urethral sphincter and bladder activity during micturition in the intact and spinally transected adult rat. Experimental Neurology, 228, 59–68. 21167152 10.1016/j.expneurol.2010.12.008

[ejn14885-bib-0014] Davies, S. N. , & Lodge, D. (1987). Evidence for involvement of N‐methylaspartate receptors in 'wind‐up' of class 2 neurones in the dorsal horn of the rat. Brain Research, 424, 402–406. 10.1016/0006-8993(87)91487-9 2823998

[ejn14885-bib-0015] Davies, S. N. , Martin, D. , Millar, J. D. , Aram, J. A. , Church, J. , & Lodge, D. (1988). Differences in results from in vivo and in vitro studies on the use‐dependency of N‐methylaspartate antagonism by MK‐801 and other phencyclidine receptor ligands. European Journal of Pharmacology, 145, 141–151. 10.1016/0014-2999(88)90225-7 2832187

[ejn14885-bib-0016] Debanne, D. , Gähwiler, B. H. , & Thompson, S. M. (1994). Asynchronous pre‐ and postsynaptic activity induces associative long‐term depression in area CA1 of the rat hippocampus in vitro. Proceedings of the National Academy of Sciences of the United States of America, 91, 1148–1152. 7905631 10.1073/pnas.91.3.1148 PMC521471

[ejn14885-bib-0017] Deng, Z. , Lisanby, S. , & Peterchev, A. (2013). Electric field depth–focality tradeoff in transcranial magnetic stimulation: Simulation comparison of 50 coil designs. Brain Stimulation, 6, 1–13. 10.1016/j.brs.2012.02.005 22483681 PMC3568257

[ejn14885-bib-0018] Dickenson, A. H. , & Sullivan, A. F. (1987). Evidence for a role of the NMDA receptor in the frequency dependent potentiation of deep rat dorsal horn nociceptive neurones following C fibre stimulation. Neuropharmacology, 26, 1235–1238. 10.1016/0028-3908(87)90275-9 2821443

[ejn14885-bib-0019] Efthimiadis, K. G. , Samaras, T. , & Polyzoidis, K. S. (2010). Magnetic stimulation of the spine: The role of tissues and their modelling. Physics in Medicine & Biology, 55, 2541–2553. 20393231 10.1088/0031-9155/55/9/008

[ejn14885-bib-0020] Formento, E. , Minassian, K. , Wagner, F. , Mignardot, J. B. , Le Goff‐Mignardot, C. G. , Rowald, A. , … Courtine, G. (2018). Electrical spinal cord stimulation must preserve proprioception to enable locomotion in humans with spinal cord injury. Nature Neuroscience, 21, 1728–1741. 30382196 10.1038/s41593-018-0262-6 PMC6268129

[ejn14885-bib-0021] Fujishiro, T. , Takahashi, S. , Enomoto, H. , Ugawa, Y. , Ueno, S. , & Kitamura, T. (2002). Magnetic stimulation of the sacral roots for the treatment of urinary frequency and urge incontinence: An investigational study and placebo controlled trial. Journal of Urology, 168, 1036–1039. 10.1016/S0022-5347(05)64569-7 12187217

[ejn14885-bib-0022] García‐Alías, G. , Petrosyan, H. A. , Schnell, L. , Horner, P. J. , Bowers, W. J. , Mendell, L. M. , … Arvanian, V. L. (2011). Chondroitinase ABC combined with neurotrophin NT‐3 secretion and NR2D expression promotes axonal plasticity and functional recovery in rats with lateral hemisection of the spinal cord. Journal of Neuroscience, 31, 17788–17799. 10.1523/JNEUROSCI.4308-11.2011 22159095 PMC3758578

[ejn14885-bib-0023] Gerasimenko, Y. , Gorodnichev, R. , Machueva, E. , Pivovarova, E. , Semyenov, D. , Savochin, A. , … Edgerton, V. R. (2010). Novel and direct access to the human locomotor spinal circuitry. Journal of Neuroscience, 30, 3700–3708. 20220003 10.1523/JNEUROSCI.4751-09.2010 PMC2847395

[ejn14885-bib-0024] Hayashi, Y. , Shi, S. H. , Esteban, J. A. , Piccini, A. , Poncer, J. C. , & Malinow, R. (2000). Driving AMPA receptors into synapses by LTP and CaMKII: Requirement for GluR1 and PDZ domain interaction. Science, 287, 2262–2267. 10.1126/science.287.5461.2262 10731148

[ejn14885-bib-0025] Ho, S. M. , & Waite, P. M. (2002). Effects of different anesthetics on the paired‐pulse depression of the h reflex in adult rat. Experimental Neurology, 177, 494–502. 12429194 10.1006/exnr.2002.8013

[ejn14885-bib-0026] Hofstoetter, U. S. , Danner, S. M. , Freundl, B. , Binder, H. , Mayr, W. , Rattay, F. , & Minassian, K. (2015). Periodic modulation of repetitively elicited monosynaptic reflexes of the human lumbosacral spinal cord. Journal of Neurophysiology, 1141, 400–410. 10.1152/jn.00136.2015 PMC4507962 25904708

[ejn14885-bib-0027] Hofstoetter, U. S. , Freundl, B. , Binder, H. , & Minassian, K. (2018). Common neural structures activated by epidural and transcutaneous lumbar spinal cord stimulation: Elicitation of posterior root‐muscle reflexes. PLoS One, 13, e0192013 10.1371/journal.pone.0192013 29381748 PMC5790266

[ejn14885-bib-0028] Hou, J. , Nelson, R. , Nissim, N. , Parmer, R. , Thompson, F. J. , & Bose, P. (2014). Effect of combined treadmill training and magnetic stimulation on spasticity and gait impairments after cervical spinal cord injury. Journal of Neurotrauma, 31, 1088–1106. 10.1089/neu.2013.3096 24552465

[ejn14885-bib-0029] Hunanyan, A. S. , Alessi, V. , Patel, S. , Pearse, D. D. , Matthews, G. , & Arvanian, V. L. (2011). Alterations of action potentials and the localization of Nav1.6 sodium channels in spared axons after hemisection injury of the spinal cord in adult rats. Journal of Neurophysiology, 105, 1033–1044. 10.1152/jn.00810.2010 21177993 PMC3074424

[ejn14885-bib-0030] Hunanyan, A. S. , Petrosyan, H. A. , Alessi, V. , & Arvanian, V. L. (2012). Repetitive spinal electromagnetic stimulation opens a window of synaptic plasticity in damaged spinal cord: Role of NMDA receptors. Journal of Neurophysiology, 107, 3027–3039. 10.1152/jn.00015.2012 22402659

[ejn14885-bib-0031] James, N. D. , Bartus, K. , Grist, J. , Bennett, D. L. , McMahon, S. B. , & Bradbury, E. J. (2011). Conduction failure following spinal cord injury: Functional and anatomical changes from acute to chronic stages. Journal of Neuroscience, 31, 18543–18555. 10.1523/JNEUROSCI.4306-11.2011 22171053 PMC3495307

[ejn14885-bib-0032] Jankowska, E. , Kaczmarek, D. , Bolzoni, F. , & Hammar, I. (2017). Long‐lasting increase in axonal excitability after epidurally applied DC. Journal of Neurophysiology, 118, 1210–1220. 28515284 10.1152/jn.00148.2017 PMC5547254

[ejn14885-bib-0033] Jin, J. , Gong, K. , Zou, X. , Wang, R. , Lin, Q. , & Chen, J. (2013). The blockade of NMDA receptor ion channels by ketamine is enhanced in developing rat cortical neurons. Neuroscience Letters, 539, 11–15. 23395831 10.1016/j.neulet.2013.01.034 PMC3602117

[ejn14885-bib-0034] Knikou, M. (2008). The H‐reflex as a probe: Pathways and pitfalls. Journal of Neuroscience Methods, 171, 1–12. 18394711 10.1016/j.jneumeth.2008.02.012

[ejn14885-bib-0035] Knikou, M. (2013). Neurophysiological characteristics of human leg muscle action potentials evoked by transcutaneous magnetic stimulation of the Spine. Bioelectromagnetics, 34, 200–210. 10.1002/bem.21768 23192827

[ejn14885-bib-0036] Knikou, M. , & Murray, L. M. (2019). Repeated transspinal stimulation decreases soleus H‐reflex excitability and restores spinal inhibition in human spinal cord injury. PLoS One, 14(9). 10.1371/journal.pone.0223135 PMC6762874 31557238

[ejn14885-bib-0037] Kocsis, J. D. , & Waxman, S. G. (1982). Intra‐axonal recordings in rat dorsal column axons: Membrane hyperpolarization and decreased excitability precede the primary afferent depolarization. Brain Research, 238, 222–227. 10.1016/0006-8993(82)90787-9 6282392

[ejn14885-bib-0038] Krause, P. , Edrich, T. , & Straube, A. (2004). Lumbar repetitive magnetic stimulation reduces spastic tone increase of the lower limbs. Spinal Cord, 42, 67–72. 10.1038/sj.sc.3101564 14765138

[ejn14885-bib-0039] Kunesch, E. , Knecht, S. , Classen, J. , Roick, H. , Tyercha, C. , & Benecke, R. (1993). Somatosensory evoked potentials (SEPs) elicited by magnetic nerve stimulation. Electroencephalography and Clinical Neurophysiology, 88, 459–467. 7694832 10.1016/0168-5597(93)90035-n

[ejn14885-bib-0040] Ladenbauer, J. , Minassian, K. , Hofstoetter, U. S. , Dimitrijevic, M. R. , & Rattay, F. (2010). Stimulation of the human lumbar spinal cord with implanted and surface electrodes: A computer simulation study. IEEE Transactions on Neural Systems and Rehabilitation Engineering, 18, 637–645. 21138794 10.1109/TNSRE.2010.2054112

[ejn14885-bib-0041] Lee, J. K. , Emch, G. S. , Johnson, C. S. , & Wrathall, J. R. (2005). Effect of spinal cord injury severity on alterations of the H‐reflex. Experimental Neurology, 196, 430–440. 16185689 10.1016/j.expneurol.2005.08.018

[ejn14885-bib-0042] Lee, J. K. , Johnson, C. S. , & Wrathall, J. R. (2007). Up‐regulation of 5‐HT2 receptors is involved in the increased H‐reflex amplitude after contusive spinal cord injury. Experimental Neurology, 203, 502–511. 17059818 10.1016/j.expneurol.2006.09.003 PMC1859857

[ejn14885-bib-0043] Lee‐Kubli, C. A. , & Calcutt, N. A. (2014). Altered rate‐dependent depression of the spinal H‐reflex as an indicator of spinal disinhibition in models of neuropathic pain. Pain, 155, 250–260. 10.1016/j.pain.2013.10.001 24103402 PMC3946970

[ejn14885-bib-0044] Leydeker, M. , Delva, S. , Tserlyuk, I. , Yau, J. , Wagdy, M. , Hawash, A. , … Ahmed, Z. (2013). The effects of 15 Hz trans‐spinal magnetic stimulation on locomotor control in mice with chronic contusive spinal cord injury. Electromagnetic Biology and Medicine, 32, 155–164. 10.3109/15368378.2013.776353 23675618

[ejn14885-bib-0045] Lüscher, C. , Xia, H. , Beattie, E. C. , Carroll, R. C. , von Zastrow, M. , Malenka, R. C. , & Nicoll, R. A. (1999). Role of AMPA receptor cycling in synaptic transmission and plasticity. Neuron, 24, 649–658. 10.1016/S0896-6273(00)81119-8 10595516

[ejn14885-bib-0046] MacDonald, J. F. , Bartlett, M. C. , Mody, I. , Pahapill, P. , Reynolds, J. N. , Salter, M. W. , … Pennefather, P. S. (1991). Actions of ketamine, phencyclidine and MK‐801 on NMDA receptor currents in cultured mouse hippocampal neurones. Journal of Physiology, 432, 483–508. 10.1113/jphysiol.1991.sp018396 PMC1181337 1832184

[ejn14885-bib-0047] Massé‐Alarie, H. , Beaulieu, L. D. , Preuss, R. , & Schneider, C. (2017). Repetitive peripheral magnetic neurostimulation of multifidus muscles combined with motor training influences spine motor control and chronic low back pain. Clinical Neurophysiology, 128, 442–453. 10.1016/j.clinph.2016.12.020 28160750

[ejn14885-bib-0048] Massé‐Alarie, H. , Flamand, V. H. , Moffet, H. , & Schneider, C. (2013). Peripheral neurostimulation and specific motor training of deep abdominal muscles improve posturomotor control in chronic low back pain. Clinical Journal of Pain, 29, 814–823. 10.1097/AJP.0b013e318276a058 23370067

[ejn14885-bib-0049] Matsumoto, L. , Hanajima, R. , Matsumoto, H. , Ohminami, S. , Terao, Y. , Tsuji, S. , & Ugawa, Y. (2010). Supramaximal responses can be elicited in hand muscles by magnetic stimulation of the cervical motor roots. Brain Stimulation, 3, 153–160. 20633444 10.1016/j.brs.2009.09.001

[ejn14885-bib-0050] Metz, G. A. , Curt, A. , van de Meent, H. , Klusman, I. , Schwab, M. E. , & Dietz, V. (2000). Validation of the weight‐drop contusion model in rats: A comparative study of human spinal cord injury. Journal of Neurotrauma, 17, 1–17. 10.1089/neu.2000.17.1 10674754

[ejn14885-bib-0051] Mezzarane, R. A. , & Kohn, A. F. (2002). Bilateral soleus H‐reflexes in humans elicited by simultaneous trains of stimuli: Symmetry, variability, and covariance. Journal of Neurophysiology, 87, 2074–2083. 10.1152/jn.00129.2001 11929925

[ejn14885-bib-0052] Minassian, K. , Persy, I. , Rattay, F. , Pinter, M. M. , Kern, H. , & Dimitrijevic, M. R. (2007). Human lumbar cord circuitries can be activated by extrinsic tonic input to generate locomotor‐like activity. Human movement science, 26, 275–295. 17343947 10.1016/j.humov.2007.01.005

[ejn14885-bib-0053] Nevian, T. , & Sakmann, B. (2004). Single spine Ca2+ signals evoked by coincident EPSPs and backpropagating action potentials in spiny stellate cells of layer 4 in the juvenile rat somatosensory barrel cortex. Journal of Neuroscience, 24, 1689–1699. 10.1523/JNEUROSCI.3332-03.2004 14973235 PMC6730461

[ejn14885-bib-0054] Nielsen, J. F. , Sinkjaer, T. , & Jakobsen, J. (1996). Treatment of spasticity with repetitive magnetic stimulation; a double‐blind placebo‐controlled study. Multiple sclerosis, 2, 227–232. 9050361 10.1177/135245859600200503

[ejn14885-bib-0055] Niu, T. , Bennett, C. J. , Keller, T. L. , Leiter, J. C. , & Lu, D. C. (2018). A proof‐of‐Concept study of transcutaneous magnetic spinal cord stimulation for neurogenic bladder. Scientific Reports, 8, 12549. 30135433 10.1038/s41598-018-30232-z PMC6105631

[ejn14885-bib-0056] Palmieri, R. M. , Ingersoll, C. D. , & Hoffman, M. A. (2004). The hoffmann reflex: Methodologic considerations and applications for use in sports medicine and athletic training research. Journal of Athletic Training, 39, 268–277. 16558683 PMC522151

[ejn14885-bib-0057] Petersen, J. A. , Spiess, M. , Curt, A. , Dietz, V. , Schubert, M. , & EM‐SCI Study Group (2012). Spinal cord injury: one‐year evolution of motor‐evoked potentials and recovery of leg motor function in 255 patients. Neurorehabilitation and neural repair, 26, 939–948. 22460611 10.1177/1545968312438437

[ejn14885-bib-0058] Petrosyan, H. A. , Alessi, V. , Hunanyan, A. S. , Sisto, S. A. , & Arvanian, V. L. (2015). Spinal electro‐magnetic stimulation combined with transgene delivery of neurotrophin NT‐3 and exercise: Novel combination therapy for spinal contusion injury. Journal of Neurophysiology, 114, 2923–2940. 10.1152/jn.00480.2015 26424579 PMC4737407

[ejn14885-bib-0059] Petrosyan, H. A. , Alessi, V. , Singh, V. , Hunanyan, A. S. , Levine, J. M. , & Arvanian, V. L. (2014). Transduction efficiency of neurons and glial cells by AAV‐1, ‐5, ‐9, ‐rh10 and ‐hu11 serotypes in rat spinal cord following contusion injury. Gene Therapy, 21, 991–1000. 10.1038/gt.2014.74 25119378

[ejn14885-bib-0060] Petrosyan, H. A. , Alessi, V. , Sisto, S. A. , Kaufman, M. , & Arvanian, V. L. (2017). Transcranial magnetic stimulation (TMS) responses elicited in hind limb muscles as an assessment of synaptic plasticity in spino‐muscular circuitry after chronic spinal cord injury. Neuroscience Letters, 642, 37–42. 28159637 10.1016/j.neulet.2017.01.065

[ejn14885-bib-0061] Petrosyan, H. A. , Fahmy, M. , Tesfa, A. , Liang, L. , & Arvanian, V. (2019) [661.08/H35] Spinal electromagnetic stimulation results in immediate pain reduction and induces long‐lasting functional improvements in patients with chronic low back pain (LBP). A pilot study. Neuroscience Meeting Planner. Society for Neuroscience, Chicago, IL.

[ejn14885-bib-0062] Rattay, F. , Minassian, K. , & Dimitrijevic, M. R. (2000). Epidural electrical stimulation of posterior structures of the human lumbosacral cord: 2. quantitative analysis by computer modeling. Spinal Cord, 38, 473–489. 10.1038/sj.sc.3101039 10962608

[ejn14885-bib-0063] Reese, N. B. , Skinner, R. D. , Mitchell, D. , Yates, C. , Barnes, C. N. , Kiser, T. S. , & Garcia‐Rill, E. (2006). Restoration of frequency‐dependent depression of the H‐reflex by passive exercise in spinal rats. Spinal Cord, 44, 28–34. 10.1038/sj.sc.3101810 16044168

[ejn14885-bib-0064] Sasada, S. , Kato, K. , Kadowaki, S. , Groiss, S. J. , Ugawa, Y. , Komiyama, T. , & Nishimura, Y. (2014). Volitional walking via upper limb muscle‐controlled stimulation of the lumbar locomotor center in man. Journal of Neuroscience, 34, 11131–11142. 25122909 10.1523/JNEUROSCI.4674-13.2014 PMC6705266

[ejn14885-bib-0065] Sayenko, D. G. , Angeli, C. , Harkema, S. J. , Edgerton, V. R. , & Gerasimenko, Y. P. (2014). Neuromodulation of evoked muscle potentials induced by epidural spinal‐cord stimulation in paralyzed individuals. Journal of Neurophysiology, 111, 1088–1099. 24335213 10.1152/jn.00489.2013 PMC3949232

[ejn14885-bib-0066] Scheff, S. W. , Rabchevsky, A. G. , Fugaccia, I. , Main, J. A. , & Lumpp, J. E. Jr (2003). Experimental modeling of spinal cord injury: Characterization of a force‐defined injury device. Journal of Neurotrauma, 20, 179–193. 10.1089/08977150360547099 12675971

[ejn14885-bib-0067] Schindler‐Ivens, S. , & Shields, R. K. (2000). Low frequency depression of H‐reflexes in humans with acute and chronic spinal‐cord injury. Experimental Brain Research, 133, 233–241. 10968224 10.1007/s002210000377 PMC4034370

[ejn14885-bib-0068] Shanthanelson, M. , Arvanian, V. L. , & Mendell, L. M. (2009). Input‐specific plasticity of N‐methyl‐D‐aspartate receptor‐mediated synaptic responses in neonatal rat motoneurons. European Journal of Neuroscience, 29, 2125–2136. 10.1111/j.1460-9568.2009.06769.x PMC2931593 19490018

[ejn14885-bib-0069] Struppler, A. , Binkofski, F. , Angerer, B. , Bernhardt, M. , Spiegel, S. , Drzezga, A. , & Bartenstein, P. (2007). A fronto‐parietal network is mediating improvement of motor function related to repetitive peripheral magnetic stimulation: A PET‐H2O15 study. NeuroImage, 36, T174–T186. 10.1016/j.neuroimage.2007.03.033 17499165

[ejn14885-bib-0070] Thompson, A. K. , & Wolpaw, J. R. (2015). Restoring walking after spinal cord injury: Operant conditioning of spinal reflexes can help. Neuroscientist, 21, 203–215. 10.1177/1073858414527541 24636954 PMC4167198

[ejn14885-bib-0071] Thompson, F. J. , Parmer, R. , & Reier, P. J. (1998). Alteration in rate modulation of reflexes to lumbar motoneurons after mid thoracic spinal cord injury in the rat. I. Contusion Injury. Journal of Neurotrauma, 15, 495–508. 10.1089/neu.1998.15.495 9674553

[ejn14885-bib-0072] Thompson, F. J. , Reier, P. J. , Lucas, C. C. , & Parme, R. (1992). Altered patterns of reflex excitability subsequent to contusion injury of the rat spinal cord. Journal of Neurophysiology, 68, 1473–1486. 1479425 10.1152/jn.1992.68.5.1473

[ejn14885-bib-0073] Tsai, P. Y. , Wang, C. P. , Chiu, F. Y. , Tsai, Y. A. , Chang, Y. C. , & Chuang, T. Y. (2009). Efficacy of functional magnetic stimulation in neurogenic bowel dysfunction after spinal cord injury. Journal of Rehabilitation Medicine, 41, 41–47. 19197568 10.2340/16501977-0280

[ejn14885-bib-0074] Ugawa, Y. , Rothwell, J. C. , & Day, B. L. (1989). Thompson PD, Marsden CD. Magnetic stimulation over the spinal enlargements. Journal of Neurology, Neurosurgery and Psychiatry, 52, 1025–1032. 10.1136/jnnp.52.9.1025 PMC1031736 2795071

[ejn14885-bib-0075] Väisänen, J. , Lindén, A. M. , Lakso, M. , Wong, G. , Heinemann, U. , & Castrén, E. (1999). Excitatory actions of NMDA receptor antagonists in rat entorhinal cortex and cultured entorhinal cortical neurons. Neuropsychopharmacology, 21, 137–146. 10.1016/S0893-133X(99)00006-8 10379528

[ejn14885-bib-0076] Xue, Y. , Han, X. H. , & Chen, L. (2010). Effects of pharmacological block of GABA(A) receptors on pallidal neurons in normal and Parkinsonian state. Frontiers Cellular Neuroscience, 4, 2. 10.3389/neuro.03.002.2010 PMC2831626 20204138

[ejn14885-bib-0077] Yamanishi, T. , Sakakibara, R. , Uchiyama, T. , Suda, S. , Hattori, T. , Ito, H. , & Yasuda, K. (2000). Comparative study of the effects of magnetic versus electrical stimulation on inhibition of detrusor overactivity. Urology, 56, 777–781. 10.1016/S0090-4295(00)00779-2 11068300

[ejn14885-bib-0078] Yang, X. C. , & Reis, D. J. (1999). Agmatine selectively blocks the N‐methyl‐D‐aspartate subclass of glutamate receptor channels in rat hippocampal neurons. Journal of Pharmacology and Experimental Therapeutics, 288, 544–549. 9918557

